# Neuropathic-like pain affects pain perception in patients with deep endometriosis: an observational study

**DOI:** 10.1007/s00404-025-08178-1

**Published:** 2025-09-10

**Authors:** Carolina Dolci, Estelle Jean dit Gautier, Lucie Lannez, Gilles Lebuffe, Jean Michel Wattier, Chrystele Rubod

**Affiliations:** 1 Departement of Gynecology, CHU Lille, Av. Eugène Avinée, 59000 Lille, France; 2 Faculty of Medicine, University of Lille, 59000 Lille, France

**Keywords:** Endometriosis, Neuropathic pain, Pain management, Catastrophizing

## Abstract

**Purpose:**

Endometriosis is a chronic, hormone-dependent disease affecting up to 10% of women of reproductive age, often associated with chronic pelvic pain (CPP). Neuropathic pain has been increasingly recognized as a significant component in a subset of patients with CPP related to endometriosis. The study objective was to assess the prevalence of neuropathic-like pain in women with deep endometriosis (DE) and CPP, and to analyze its influence on pain perception and quality of life.

**Methods:**

Retrospective monocentric cohort study included 149 women with DE and CPP treated at a tertiary pain center between 2013 and 2017. Pain characteristics were assessed using validated tools, including the DN4 questionnaire for neuropathic pain, the abridged Saint-Antoine Pain Questionnaire (QDSA) for sensory and emotional dimensions of pain, and the EQ-VAS for quality of life. Psychological factors, including anxiety, depression, and catastrophizing, were also evaluated.

**Results:**

Neuropathic-like pain was identified in 36% of patients. These patients reported significantly higher global and minimum pain intensity (*p* < 0.01, *p* < 0.01), greater emotional (QDSA affective subscore, *p* < 0.05) and sensory (QDSA sensory subscore, *p* < 0.001) pain impact, and higher catastrophizing scores (*p* < 0.001). Quality of life was notably impaired (*p* < 0.05). Neuropathic-like pain was not associated with the stage of endometriosis or surgical complexity. Anxiety and depression scores did not differ significantly between the two groups (*p* = 0.47 and *p* = 0.52, respectively).

**Conclusions:**

Neuropathic-like pain was retrieved in over one-third of patients with DE and CPP, contributing to greater pain intensity, emotional distress, and reduced quality of life. Systematic screening for neuropathic-like pain and tailored multidisciplinary care are essential to optimize pain management.

## Introduction

Endometriosis, defined as the presence of endometrium-like tissue outside the uterus, is a benign, chronic, hormone-dependent disease, affecting up to 10% of women in reproductive age [1].

The etiopathogenesis of endometriosis involves multifactorial processes. A combination of genetic, epigenetic, immunological, inflammatory, and hormonal factors is involved, resulting in a heterogeneous disease [2].

Endometriosis-related pain includes dysmenorrhea, dyschezia, dysuria, dyspareunia, and chronic pelvic pain (CPP) [3, 4]. Deep endometriosis (DE) involves lesions infiltrating > 5 mm beneath the peritoneum, often affecting the uterosacral ligaments, rectovaginal septum, bladder, and bowel. DE is linked to chronic, severe pain and higher risk of central sensitization and emotional comorbidities [5, 6], though lesion extent does not linearly predict pain intensity [7, 8].

CPP, defined as intermittent or continuous lower abdominal pain for at least 6 months that causes functional disability and is not due to menstruation or intercourse [9], is reported in over 60% of endometriosis patients [10]. However, there is growing recognition that severe dysmenorrhea may itself fall within the broader CPP category, as it often leads to central sensitization and functional impairment [11].

Endometriosis-related pain was historically considered predominantly nociceptive, linked to local inflammation. However, recent studies highlight the involvement of nociceptive, neuropathic, and nociplastic mechanisms [12, 13]. Neuropathic pain results from somatosensory system damage, while neuropathic-like pain mimics such features without confirmed injury [14]. In a recent study on chronic pelvic pain, over 50% of endometriosis patients (*n* = 32) exhibited neuropathic features [15]. Endometriosis-related neuropathic pain could be expected to arise in the context of endometriosis for a number of reasons, such as the possible nerve damage during surgery, the possible nerve involvement from the endometriotic lesion [16], and inflammatory mediators such as Brain-Derived Neurotrophic Factor (BDNF) and Tumor Necrosis Factor (TNF)-alpha in women with endometriosis, potentially sensitizing nerve endings [17].

There is large evidence from both animal and human studies showing that neuropathic and nociceptive pain differs considerably, including with respect to their underlying pathology [18, 19], cognitive-affective processing [20, 21], and responses to treatment. Therefore, a better understanding of the prevalence of neuropathic pain in endometriosis could guide both clinical care and future clinically oriented research strategies.

The objective of this study, therefore, was to assess the prevalence of neuropathic-like pain in women with DE and to explore its association with pain perception, emotional burden, catastrophizing, and quality of life, to better characterize the multidimensional nature of pain in this population.

## Methods

We carried out a monocentric retrospective observational cohort study from January 2013 to December 2017. We included women with DE [22] and CPP addressed at the Pain Center of the Anesthesia and Resuscitation Departement of Claude Huriez Hospital in Lille, France. Both patients with histologically confirmed endometriosis and patients with suspected endometriosis, basing on gynecological examination and imaging (transvaginal ultrasound or/and magnetic resonance imaging), were included.

Patients suffering from isolated adenomyosis, other gynecological or gastro-intestinal conditions, other chronic disease, immunological disease, or with a history of non-endometriotic pelvic surgery and previous pelvic radiotherapy were excluded from the study.

For each included patient, general characteristics such as age, parity, medical history, history of infertility, previous use of assisted reproductive technique (ART), previous surgeries for endometriosis, American Fertility Society score (AFSr score) at the time of surgery were retrieved from patients medical records [23].

At the time of the first consultation, all pain characteristics were collected and several assessments for the evaluation of all different dimensions of pain were conducted.

The intensity of pain was assessed using the numerical rating scale (NRS) rating from 0 to 10: The pain was considered of low intensity for 0–3 values, moderate for 4–7 values, and severe for 8–10 values [24]. Patients were asked to rate: (1) average pain over the past week (global), (2) the most intense pain episode (maximum), and (3) the least intense moment (minimum), in order to capture intra-individual variability and reflect the fluctuating nature of pain in deep endometriosis.

Pain characteristics—such as duration, frequency, and aggravating factors (e.g., menstruation, urination, intercourse, defecation)—were collected via standardized interviews. Patients reported the average duration of typical pain flares. Variables were reported as frequencies and selected for their relevance in DE, where pelvic involvement and hypersensitivity may contribute to neuropathic-like pain.

To evaluate the presence of neuropathic-like pain, the patients underwent the “Neuropathic Pain in 4 parts (DN4)” assessment developed by Bouhassira et al. in 2005 [25]. It consists of two parts: The first part (DN4 interview) concerns the characteristics of pain (burns, painful sensation of cold and electric shocks), and the association with paresthesia or dysesthesia (tingling, tingling, numbness, itching); a score equal or greater than 4 was considered positive. The second part corresponds to the clinical examination consisting in the assessment of the presence of hypoesthesia with tact or sting and allodynia. The overall score is 10 and a score equal or greater than 4 was considered positive, with a sensitivity of 82.9% and a specificity of 89.9%, with a Cronbach’s alpha values between 0.67 and 0.74 [26].

For this study, we used the DN4 interview component alone to categorize patients, applying a cutoff of ≥ 3, which has demonstrated good discriminative validity in previous clinical and epidemiological studies [25]. Based on this, the study population was divided into two groups: patients with DN4 interview score ≥ 3 (neuropathic-like pain) and < 3 (non-neuropathic-like pain).

To assess the sensory and emotional dimension of pain, the abridged Saint-Antoine Pain Questionnaire (QDSA), a French adaptation of the McGill Pain Questionnaire [27], was then administered. It includes 16 items: The first nine items investigate the sensory side of pain (stinging, penetrating, electric shock, stabs, vise, tugging, burning, tingling, and heaviness) and the last seven items investigate the emotional side of the pain (exhausting, scary, haunting, unbearable, annoying, maddening, and depressing). Example items include: “Does your pain feel like a stabbing sensation?” (sensory) or “Does your pain feel exhausting or depressing?” (emotional). Each item is rated from 0 (absent) to 4 (extremely strong). The sensory subtotal score is from 0 to 36, and the affective subtotal score is from 0 to 28. The total score for the abbreviated QDSA is between 0 and 64. The abridged QDSA has shown acceptable internal consistency in previous studies (Cronbach’s *α* ≈ 0.75) [28].

Sleep disturbances, assessed through standardized questions, were included due to their known association with chronic pelvic pain and their impact on pain perception and quality of life.

In order to assess the anxiety and depressive context linked to chronic pain symptoms, the "Hospital Anxiety and Depression" (HAD) questionnaire was used [29, 30]. It consists of seven questions (rating 0 to 3) assessing anxiety and seven questions assessing depression. Items refer to the emotional state over the past week, such as “I feel tense or ‘wound up’” (anxiety) or “I have lost interest in my appearance” (depression). The maximum score for each part is 21, with a positive threshold of 11/21. In combination, the abridged Beck questionnaire made it possible to qualify depressive symptoms. Example items include: “I feel sad” or “I have lost interest in people.” It is made up of 13 items ranging from 0 to 3. A score between 4 and 7 indicates a mild depression, a score between 8 and 15 a moderate depression, and a score greater than 16 a severe depression.

Catastrophism, defined by an excessively negative behavioral and affective response, was assessed by the Pain Catastrophizing Scale containing 13 items [31, 32]. Items prompt reflection on thoughts during pain, such as: “I worry all the time about whether the pain will end” or “I keep thinking of other painful events.” Each item is rated from 0 (not at all) to 4 (permanently). The PCS has demonstrated excellent internal consistency across populations (Cronbach’s *α* = 0.92) [33].

In the end, the patient's health status was assessed by a self-questionnaire, the EQ-VAS. It is a 20 cm graduated scale from 0 to 100. The score of 100 represents the best health status, and 0 represents the worst health status.

Statistical analysis was carried out using the BiostaTGV software and was validated by the public health department of the Lille University Hospital. The questionnaire responses were grouped into categorical variables (e.g., pain intensity levels, presence/absence of specific symptoms) and compared using Fisher’s exact test or the Chi-square test, as appropriate. Qualitative variables were described in frequency (percentage). The quantitative variables were compared by a Mann–Whitney test. The quantitative variables were expressed as an average (± Standard Deviation). A p-value of 0.05 was chosen as the statistical significance threshold.

### Institutional review board approval

These were volunteer patients, and an agreement from the CNIL of Lille University (National Data Protection Commission: Protection of personal data) was obtained.

## Results

A total of 149 patients with pelvic endometriosis were included in the study. Fifty-four patients (36%, 95% CI: 29–44.1) suffer from neuropathic-like pain (Group 1). The general characteristics of patients are summarized in Table 1. No significant differences were found between the groups, except for parity (*Chi-square test*, *p* < 0.01), age (*Mann–Whitney test*, *p* = 0.05), and surgical radicality (*Chi-square test*, *p* < 0.05).

**Table 1:** Patients’ characteristics according to neuropathic character of pain in patients assessed by the «DN4 interview» questionnaire

Variables	Population	DN4 ≥ 3	DN4 < 3	Degree significance
	*N* = 149	*N* = 54	*N* = 95	*p*
Parity (≥ 1 child)	76 (51%)	19 (35%)	57 (60%)	< .01*
Age (years)	35,7 (± 11,4)	34,8 (± 6,9)	36,9 (± 6,4)	.05^§^
AFSr stage				
1	36 (31%)	14 (33%)	22 (31%)	1*
2	28 (24%)	11 (26%)	17 (24%)	1*
3	16 (14%)	8 (19%)	8 (11%)	.40*
4	35 (30%)	10 (23%)	24 (34%)	.29*
NS	34	11	24	-
Surgery	126 (85%)	45 (83%)	81 (85%)	.81*
Laparoscopy	123 (98%)	44 (81%)	79 (83%)	1*
Laparotomy	19 (15%)	4 (7%)	15 (16%)	.19°
Surgical procedure				
Exploration	47 (32%)	20 (37%)	26 (27%)	.18*
Complete resection of lesions	101 (68%)	31 (57%)	70 (74%)	< .05*
Pre-MAP	18 (12%)	9 (16%)	9 (9%)	.19*
Bowel resection	27 (18%)	11 (20%)	17 (18%)	.66*
Urological resection	13 (9%)	5 (9%)	8 (8%)	1°
MAP	48 (32%)	19 (35%)	29 (30%)	.57*

Results expressed in number of cases (%). Fisher’s statistical test, *p*-value < 0,05; OR CI 95%: odds ratio: confidence interval at 95%

DN4: Neuropathic Pain Questionnaire in 4 parts

Stage: according to AFSr classification, stage 1: minimal; stage 2: mild; stage 3: moderate; stage 4: severe, NS: not specified; percentages for stages I–IV were calculated based on the number of patients with available staging information (*N* = 115). The NS category refers to patients for whom rAFS staging data were not available

PE complete resection: complete resection of pelvic endometriosis lesions

MAP: medically assisted procreation

^*^Chi-square test

°Fisher exact test

^§^Mann–Whitney

The mean pain intensity was 5.34 ± 2.2 overall, with a maximum pain intensity of 8.6 ± 1.4 and a minimum pain intensity of 2.4 ± 2.2. Both global and minimum pain intensities were significantly higher in women with suspected neuropathic pain (*Mann–Whitney test*, *p* < 0.01 and *p* < 0.01, respectively).

The mean total QDSA score, the “affective” QDSA subscore, and the “sensory” QDSA subscore were all significantly higher in patients with neuropathic-like pain (*Mann–Whitney test*, *p* < 0.001, *p* < 0.05, and *p* < 0.001, respectively). No significant differences were observed between the two groups in the HAD and Beck scores. However, patients with neuropathic-like pain (Group 1) had a significantly higher mean catastrophizing score (*Mann–Whitney test*, *p* < 0.001) and significantly more impaired quality of life (*Mann–Whitney test*, *p* < 0.05) (Table 2).

**Table 2:** Quantitative variables of pain: patient pain assessment according to neuropathic character of pain in patients assessed by the « DN4 interview» questionnaire

Variables	Population	DN4 ≥ 3	DN4 < 3	*p*
	*N* = 149	*N* = 54	*N* = 95	
NRS (/10)				
Normal	5 .3 (± 2.2)	6.0 (± 2.2)	4.9 (± 2.1)	** < .01**^**§**^
Max	8.6 (± 1.4)	8.9 (± 1.1)	8.4 (± 1.5)	.14^§^
Min	2.4 (± 2.2)	3.2 (± 2.6)	1.9 (± 1.8)	** < .01**^**§**^
QDSA				
Sensory	13.7 (± 6.8)	18.5 (± 6.1)	10.8 (± 5.4)	** < .001**^**§**^
Affective	14.4 (± 7.6)	16.2 (± 7.5)	13.3 (± 7.8)	** < .05**^**§**^
Total	28.1 (± 12.8)	34.7 (± 12.2)	24.1 (± 11.5)	** < .001**^**§**^
HAD				
Anxiety (/21)	6.8 (± 4.2)	7.1 (± 4.3)	6.6 (± 4.1)	.50^**§**^
Depression (/21)	9.7 (± 4.3)	9.2 (± 4.0)	10.1 (± 4.5)	.40^**§**^
Total (/42)	16.7 (± 7.5)	16.1(± 7.3)	16.9 (± 7.6)	.46^**§**^
Beck/39	9.04 (± 5.9)	9.97 (± 6.0)	8.60 (± 5.8)	.21^**§**^
Catastrophizing/52	22.8 (± 14.1)	30.15 (± 13.06)	19.31 (± 13.3)	** < .001**^**§**^
EqVAS/100	48.6 (± 21.7)	40.4 (± 20.5)	52.7 (± 21.3)	** < .05**^**§**^

Results expressed as means (± standard deviation); Mann–Whitney statistical test, *p*-value < 0,05

DN4 interview Neuropathic Pain Questionnaire in 4 parts

NRS: numerical rating scale; QDSA: Saint-Antoine Pain Questionnaire; HAD: Hospital Anxiety and Depression; EqVAS: Visual Analogue Scale in the EQ-5D

^*^Chi-square test

°Fisher exact test

^§^Mann–Whitney

The qualitative assessment of pain is summarized in Tables 3 and 4. Patients with neuropathic-like pain were significantly more likely to report uninterrupted pain (47.2% vs. 25.5%, *p* < 0.05, Chi-square test) and to have experienced pain for approximately one year (32% vs. 15.9%, *p* < 0.05, Chi-square test), suggesting a more persistent and chronic pain pattern in this group. While most patients (61.5%) reported having pain for several years, this was not significantly different between groups (*p* = 0.07, Chi-square test).

**Table 3:** Qualitative variables of pain: pain temporality analysis (duration and frequency) according to neuropathic character of pain in patients assessed by the « DN4 interview» questionnaire

Variables	Population	DN4 ≥ 3	DN4 < 3	OR	Degree significance
	*N* = 149	*N* = 54	*N* = 95	IC 95%	*p*
Duration					
A few days	0	0	0	–	–
A few weeks	1 (0.7%)	0	1 (1%)	–	1°
A few months	5 (4%)	2 (%)	3 (3.2%)	1.18 [0.10;10.63]	1°
Six months	19 (12.8%)	6 (11.3%)	13 (13.8%)	0.80 [0.23;2.43]	0.80*
One year	32 (21.6%)	17 (32%)	15 (15.9%)	2.47 [1.03; 5.98]	< .05*
A few years	91 (61.5%)	27 (50.9%)	63 (67%)	0.51 [0.24;1.08]	0.07*
Frequency					
Chronic pain	49 (33.1%)	25 (47.2%)	24 (25.5%)	2.59 [1.20;5.63]	< 0.05*
Daily (1–2 times/day)	1 (0.7%)	0	1 (1%)	*	*
Daily (more than 2 times/day)	41 (27.7%)	15 (28.3%)	26 (27.6%)	1.03 [0.45; 2.31]	1*
Weekly (1–2 times/week)	3 (2%)	0	3 (3.2%)	*	0.55°
Several times per week	14 (9.5%)	4 (7.5%)	10 (10.6%)	0.68 [0.15;2.52]	0.77°
< 1/week	1 (0.7%)	1 (1.9%)	0	–	0.36°
Variable	49 (33.1%)	14 (26.4%)	34 (36.1%)	0.63 [0.28; 1.39]	0.27*

Results expressed in number of cases (%) Fischer’s statistical test, *p*-value < 0,05; OR CI 95%: odds ratio: confidence interval at 95%

DN4 interview: Neuropathic Pain Questionnaire (4 parts)

^*^Chi-square test

°Fisher exact test

^§^Mann–Whitney

**Table 4:** *Pain-aggravating factors according to neuropathic character of pain* assessed by the « DN4 interview» questionnaire

Variables	Population	DN4 ≥ 3	DN4 < 3	OR	Degree significance
	*n* = 149	*n* = 54	*n* = 95	IC 95%	*p*
Defecation	83 (58.4%)	33 (61.1%)	49 (51.6%)	1.25 [0.59; 2.65]	0.60*
Urination	39 (33.9%)	20 (37%)	18 (18.9%)	2.33 [0.98; 5.62]	< 0.05*
Sexual intercourse	106 (76.2%)	43 (79.6%)	62 (65.3%)	1.66 [0.68; 4.30]	0.30*
Menstruations	82 (71.3%)	32 (59.3%)	49 (51.6%)	0.84 [0.34; 2.11]	0.67*

Results expressed in number of cases (%) Fisher’s statistical test, *p*-value < 0,05

DN4 interview: Nueropathic Pain Questionnaire in 4 parts

OR CI 95%: odds ratio: confidence interval at 95%

^*^Chi-square test

°Fisher exact test

^§^Mann–Whitney

Pain-aggravating factors, including sexual intercourse (76.2%), menstruation (71.3%), and defecation (58.4%), were commonly reported. Urination was a significantly more frequent trigger among patients with neuropathic-like pain (p = 0.04, Chi-square test).

Sleep disturbances were common, with 42% of patients reporting night awakenings every other night. However, no significant differences were observed between groups (*Chi-square test*, all *p* > 0.05).

## Discussion

Pain, in its various forms, is the most frequent and debilitating symptom associated with endometriosis. Nociceptive pain—defined as pain arising from actual or threatened damage to non-neural tissue and mediated by nociceptors [34]—is thought to originate directly from endometriotic lesions. This may be related to the inflammatory microenvironment, peripheral neurogenesis, and even somatic driver mutations within the lesions themselves. Consequently, hormonal suppression and/or surgical excision of endometriotic lesions is generally expected to alleviate nociceptive pain symptoms [35].

However, growing evidence suggests that the chronic pain experienced by women with endometriosis is due to nerve fiber involvement in the pelvic region contributing to a neuropathic-like pain in these patients [36], defined as pain caused by disease or lesion of the somatosensory nervous system [34].

Persistent painful stimuli may induce neuroplastic changes, including central sensitization, even without clear evidence of tissue damage or somatosensory lesions, making pain management particularly challenging [12]. Central sensitization is increasingly recognized as a key mechanism in chronic pelvic pain in endometriosis [37], contributing to symptom persistence after surgery and to comorbidities such as fatigue, sleep disturbances, and mood disorders [38]. It involves long-term hyperexcitability of central nociceptive pathways [39], possibly driven by adaptive pre- and post-synaptic changes following repeated peripheral input [40].

The study results showed that more than a third of patients suffering from deep endometriosis with chronic pelvic pain suffer from neuropathic-like pain. The presence of neuropathic-like pain according to DN4 seems to be associated with a more intense perception of pain, a major emotional and sensry impact of pain, a greater presence of catastrophism, and, consequently, a worse quality of life overall. Conversely, the presence of neuropathic-like pain does not seem to be linked either to the stage of endometriotic disease, sites of lesions, or to the complexity of the surgery performed. The association between neuropathic-like pain, greater pain intensity, emotional distress, and catastrophizing aligns with mechanisms of peripheral and central sensitization. Endometriotic lesions may irritate nerve fibers, while inflammatory mediators (e.g., TNF-α, BDNF) contribute to sensitization. Persistent nociceptive input may promote central plasticity, maintaining pain even without active lesions.

Despite higher pain scores, anxiety and depression levels did not differ significantly, contrasting with prior studies. This may reflect complex interactions between pain and psychological factors, including coping strategies or treatment history.

Our findings align with those of Coxon et al. [34], who reported that ~ 40% of patients with endometriosis-related pain screened positive for neuropathic-like features using painDETECT questionnaire. Similar to our cohort, this subgroup had higher pain intensity, greater functional impairment, and elevated anxiety, depression, and cognitive symptoms. Neuropathic pain was also linked to longer pain duration and more abdominal surgeries—associations we could not confirm due to retrospective design and missing treatment data. Morassutto et al. similarly emphasized the clinical importance of identifying neuropathic and nociplastic mechanisms in endometriosis, given their link to symptom severity and psychological burden [41].

In line with observations in other neuropathic conditions, the neuropathic subgroup of endometriosis patients reported higher pain intensities, greater psychological distress and cognitive impairment. Neuropathic pain was also more likely in patients with more abdominal surgeries and a longer history of pain, indicating that endometriosis-associated pain includes a neuropathic-like component in a substantial proportion of women and potentially changing the current conceptualization of endometriosis-associated pain as nociceptive.

Currently, symptomatic endometriosis is considered as nociceptive and it is predominantly managed by gynecologists with hormonal suppression or with surgical removal of endometriosis lesions. Pain management programs are therefore recommended only when hormonal treatment or surgery has failed.

The high prevalence of neuropathic-like pain in our sample highlights the need to assess this component, particularly in patients with persistent symptoms or prior surgeries. Systematic screening with tools like the DN4 questionnaire should be integrated into routine consultations. Identifying neuropathic features may guide multidisciplinary management, including neuromodulators or psychological support, to improve individualized care in endometriosis.

Our data suggest that patients who underwent complete resection were less likely to report neuropathic-like pain. Though causality cannot be inferred, this may indicate that adequate surgical management reduces peripheral sensitization. This aligns with studies linking complete excision—especially with nerve-sparing techniques—to improved pain and reduced neuropathic features [42].

Additionally, patients with neuropathic-like pain more often reported continuous pain and long-lasting pain, suggesting central sensitization. These findings support the role of neural plasticity in persistent pain and highlight the need for multimodal, individualized treatment. To date, while there have been trials in patients with chronic pelvic pain [43, 44], none of the available neuropathic adjunctive analgesics (e.g., amitriptyline, gabapentin, etc.) have been tested in a cohort of exclusively participants with endometriosis-associated pain, despite being relatively cheap and well tolerated. Recent calls in the literature emphasize the need to test neuromodulators in endometriosis-specific populations, especially those with confirmed neuropathic phenotypes [41, 45].

Given the complex nature of the disease and the potential significant negative impact on quality of life, there has been a long-standing recognition of the need for multidisciplinary care for people with endometriosis [46]. A multidisciplinary care program based on a biopsychosocial approach including pain education, cognitive behavioral therapy, pelvic muscle physical therapy, and targeted pharmacology could be suggested for the management of endometriotic patients in which a neuropathic pain component is suspected.

The major strength of the study was the in-person recruitment and physical examination of all included patients, differently for the majority of literature made up of surveys-retrieved data, allowing to verify the diagnosis of endometriosis, patients’ characteristics, stage, or location of the disease. In addition, the use of validated clinical tools makes our results reliable and reproducible. Moreover, the study setting was a referral center for pain management.

The main limitation of this study is its retrospective design. Detailed data on previous hormonal and surgical treatments were unavailable, potentially confounding group comparisons and limiting symptom interpretation. Similarly, hormonal therapy at the time of pain assessment was not systematically recorded, despite its relevance for pain modulation. Moreover, no standardized pelvic floor evaluation was performed, which may have missed myofascial or pudendal contributions overlapping with neuropathic symptoms.

Additionally, neuropathic-like pain was classified using the DN4 interview only, due to missing physical exam data; however, this method is validated in large-scale studies.

Finally, as our sample included only patients with deep endometriosis, generalizability to broader populations with peritoneal or ovarian endometriosis is limited.

Future prospective, multicenter studies are needed to validate these findings, improve generalizability, and explore causality. Detailed treatment histories should be collected, and interventional trials testing multidisciplinary approaches or targeted therapies for neuropathic pain in endometriosis are strongly warranted.

## Conclusion

Neuropathic pain is a significant component of endometriosis-related pain, impacting pain perception, emotional well-being, and quality of life. Systematic screening for neuropathic-like pain and comprehensive multidisciplinary management could improve outcomes in affected patients. Further research is essential to validate these findings and refine treatment strategies.

## Data Availability

No datasets were generated or analyzed during the current study.
